# Intravenous to oral switch therapy in cancer patients with catheter-related bloodstream infection due to methicillin-sensitive *Staphylococcus aureus*: A single-center retrospective observational study

**DOI:** 10.1371/journal.pone.0207413

**Published:** 2018-11-29

**Authors:** Naoya Itoh, Yoshiro Hadano, Sho Saito, Michiko Myokai, Yasunobu Nakamura, Hanako Kurai

**Affiliations:** 1 Division of Infectious Diseases, Shizuoka Cancer Center Hospital, Sunto-gun, Shizuoka, Japan; 2 Department of Infection Control and Prevention, Tokyo Medical and Dental University Hospital, Bunkyo-ku, Tokyo, Japan; 3 AMR Clinical Reference Center, Disease Control and Prevention Center, National Center for Global Health and Medicine, Tokyo, Japan; 4 Department of Medical Statistics, Satista Co., Ltd, Uji-city, Kyoto, Japan; Laurentian, CANADA

## Abstract

The most common complication in cancer patients is catheter-related bloodstream infection (CRBSI), of which *Staphylococcus aureus* is a common pathogen. Although *S*. *aureus* CRBSI patients are recommended for prolonged intravenous therapy, this is often not feasible. We assessed the effectiveness of switching from intravenous to oral antimicrobial therapy in cancer patients with CRBSI due to methicillin-sensitive *S*. *aureus* (MSSA). We conducted a retrospective observational study of 60 patients at one tertiary-care cancer center between April 2005 and March 2016. Patients who received effective intravenous (IV) antibiotics for at least 10 days (IV group) were compared to the IV group of patients who had switched to effective oral (PO) antibiotics after IV treatment for at least 10 days (IV + PO group). The primary endpoint was all-cause mortality within 90 days. Univariate and propensity score-adjusted multivariate logistic regression analyses using variables likely to influence the outcomes were performed. Of the 60 patients, 32 (53.3%) and 28 (46.7%) were in the IV and IV + PO groups, respectively. The median antibiotic treatment durations in the IV and IV + PO groups were 17 (13–31) and 33 (26–52) days, respectively (p<0.001). The 90-day mortality in the IV and IV + PO groups were 53.1% (17/32) and 10.7% (3/28), respectively (p = 0.001). Univariate logistic regression model showed that the odds ratios of oral switch therapy for 90-day mortality was 0.106 (95% confidence interval [CI]: 0.027–0.423; p = 0.001). The propensity score-adjusted multivariate logistic regression model estimated the odds ratios of oral switched therapy for 90-day mortality as 0.377 (95% CI: 0.037–3.884; p = 0.413). Our results suggest that oral switch therapy was not associated with mortality in cancer patients with CRBSI due to MSSA compared with no oral switch therapy. Oral switch therapy may be a reasonable option for patients with CRBSI due to MSSA.

## Introduction

Staphylococcal infections are common and represent an important therapeutic problem, as bacterial complications frequently arise [1]. *Staphylococcus aureus* bacteremia (SAB) is a leading bloodstream infection in hospitals, with mortality rates of 10%–30% [2]. Therefore, such infections require adequate management and highly effective anti-infective treatment. Current guidelines recommend that patients with *S*. *aureus* catheter-related bloodstream infection (CRBSI) should have the infected catheter removed, and that SAB should be treated with prolonged intravenous therapy for a minimum of 14 days in those with uncomplicated disease, and 4–6 weeks in those with deep focus of infection [3, 4]. Furthermore, 4–6 weeks of antibiotic therapy should be administered to patients with active malignancy or immunosuppression [3]. However, long-term therapy mandates additional hospital stay for the patients, which is often difficult to comply with in actual clinical practice.

Patients with low risk of complications may thus benefit from an early switch to oral medication with earlier discharge and fewer intravenous therapy complications. The effectiveness of oral antimicrobial therapy in SAB was previously assessed in a multicenter randomized controlled trial; 104 patients with SAB either received oral combination of fluoroquinolone plus rifampicin or standard parenteral treatment, and the cure rates in both groups were similar (82% versus 80%, respectively), with patients on oral antibiotics being discharged earlier [5]. This study suggests that administering oral antimicrobials may be as effective as intravenous therapy. Thwaites et al. also reported the management practices of patients with SAB in the UK and Vietnam [6]. Although they found that 25% of patients received oral antimicrobials alone for more than 50% of the treatment duration, the efficacy and safety of oral therapy were not evaluated. The *Staphylococcus aureus* Bacteremia Antibiotic Treatment Options (SABATO) trial is currently ongoing to assess whether an early switch to oral medication is as safe and effective as intravenous standard therapy in SAB [7]; however, few studies have focused on the oral switch therapy in patients with CRBSI due to methicillin-sensitive *Staphylococcus aureus* (MSSA). Central venous access is necessary for cancer patients who require chemotherapy and parenteral nutrition. Additionally, cancer patients, who are often immunocompromised, are more susceptible to CRBSI [8]. Therefore, CRBSI remains the most common complication in cancer patients.

To assess the effectiveness of switching from intravenous to oral antimicrobial therapy in cancer patients with CRBSI due to MSSA, we compared between patients who received effective intravenous (IV) antibiotics for more than 10 days and patients who were switched to an effective oral (PO) antibiotics after IV treatment for at least 10 days.

## Methods and materials

### Study design and patient population

We conducted a retrospective observational study at the Shizuoka Cancer Center Hospital, a 611-bed tertiary care hospital, from April 1, 2005 to March 31, 2016. The inclusion criteria were at least one of the following: patients with MSSA-positive blood culture, patients with CRBSI caused by MSSA, and patients who received effective intravenous antibiotics for more than 10 days or switched to an effective oral antibiotic after IV treatment for at least 10 days. Duration of intravenous antibiotics was at least 10 days for comparison with oral antibiotics after IV treatment for at least 10 days. Exclusion criteria were: (1) being less than 18 years of age, (2) polymicrobial bacteremia, (3) duplicated cases, (4) duration of IV therapy was less than 10 days, (5) patient death within 2 days after the positive blood culture, and (6) patients deemed to be palliative cases (specifically, patients who did not receive any antibiotics).

The primary outcome was all-cause mortality at 90 days. The secondary outcomes were all-cause mortality within 30 days and recurrence within 30 days after treatment.

### Ethics approval and consent to participate

Ethical approval was obtained from the Shizuoka Cancer Center Hospital’s Ethics Review Board, which also deemed it unnecessary to obtain consent directly from patients, as this was a retrospective study with no intervention.

### Data collection

Data were retrieved from patients’ electronic medical records and entered into case report forms by four infectious disease (ID) doctors. Information retrieved included age, gender, microbiological data, focus of infection, place of acquisition (nosocomial, health care-associated, and community-acquired), types of neoplasm, organ transplantation, immunosuppressant therapy, glucocorticoid therapy, antineoplastic chemotherapy, total parenteral nutrition, comorbidity, type of central venous catheters (CVCs), foreign body, severity of bacteremia, secondary foci, persistent bacteremia, removal of infectious catheter, source control, reinsertion of central intravascular catheter during treatment, complications after treatment, ID consultation, received echocardiogram (transthoracic echocardiography or transesophageal echocardiography), antibiotic treatment, duration of antibiotics, recurrence, the number of days till death following positive blood culture, and infection-related death.

### Study definitions

Staphylococcal bacteremia was defined as at least one blood culture positive for *S*. *aureus* with clinical symptoms and clinically apparent signs and symptoms of sepsis [9].

CRBSI was defined as a primary bloodstream infection in patients with CVC that was present for at least 48 hours before the onset of bacteremia and had no identifiable source of infection other than the catheter. Persistent bacteremia was defined when bacteremia occurred for ≥3 days and <5 days while patients were receiving appropriate antibiotic therapy. An immunosuppressant was defined as a drug used to prevent the rejection of transplanted organs or to treat autoimmune diseases within 3 months of the onset of SAB. Glucocorticoid therapy was also defined as a dose of 25 mg prednisone/day for >1 month, or a cumulative dose of >700 mg prednisone within 3 months of the onset of SAB [10]. Antineoplastic chemotherapy was defined as any antineoplastic chemotherapy received in the 3 months prior to culture collection [11]. Nosocomial bloodstream infection was defined as a positive blood culture obtained from patients who had been hospitalized for 48 hours or longer. If a patient was transferred from another hospital, that patient’s data was included as nosocomial bloodstream infection [12]. Health care-associated bloodstream infection was defined as a positive blood culture obtained from a patient at the time of hospital admission or within 48 hours of admission if the patient fulfilled any of the following four criteria. (1) Received intravenous therapy at home; received wound care or specialized nursing care through a health care agency, family, or friends; or had self-administered intravenous medical therapy in the 30 days before the bloodstream infection. Patients who received only oxygen therapy at home were excluded. (2) Attended a hospital or hemodialysis clinic, or received intravenous chemotherapy in the 30 days before the bloodstream infection. (3) Was hospitalized in an acute care hospital for ≥2 days in the 90 days before the bloodstream infection. (4) Resided in a nursing home or long-term care facility. Community-acquired bloodstream infection was defined as a positive blood culture obtained at the time of hospital admission or within 48 hours after hospital admission in patients not fitting the health care-associated infection criteria. The severity of bacteremia was based on the Pitt Bacteremia Score [13]. Recurrence was defined as the return of *S*. *aureus* bacteremia after the documentation of negative blood cultures or clinical improvement within 30 days of completing a course of anti-staphylococcal therapy. Duration of antibiotic therapy was defined as the number of days that the patient received effective antibiotic therapy. Effective antibiotic therapy was defined as therapy wherein the patient’s isolate was susceptible to all antibiotics and appropriate dosing. Infection-related death was defined as death attributable to bacteremia if at least one of the following was present: (1) positive blood culture at the time of death; (2) persistent signs and symptoms of infection due to that microorganism at death; (3) no other apparent cause of death other than bacteremia.

### Statistical analyses

Continuous variables with non-parametric distribution were expressed as median and interquartile range (IQR) [25%, 75%] or number and rate, and were compared using Mann-Whitney U test. Categorical variables were expressed as number and rate and compared using Fisher’s exact test. Univariate and propensity score-adjusted multivariate logistic regression analyses were performed. The propensity scores were adjusted using variables likely to influence the outcomes, and the primary outcome (i.e. all-cause mortality within 90 days) was estimated using logistic regression analysis. In the analysis, missing data were excluded, and imputation was not performed. All tests were 2-tailed, and statistical significance was defined by a P value <0.05. Statistical analyses were performed with R version 3.2.4.

## Results

**Fig 1:**
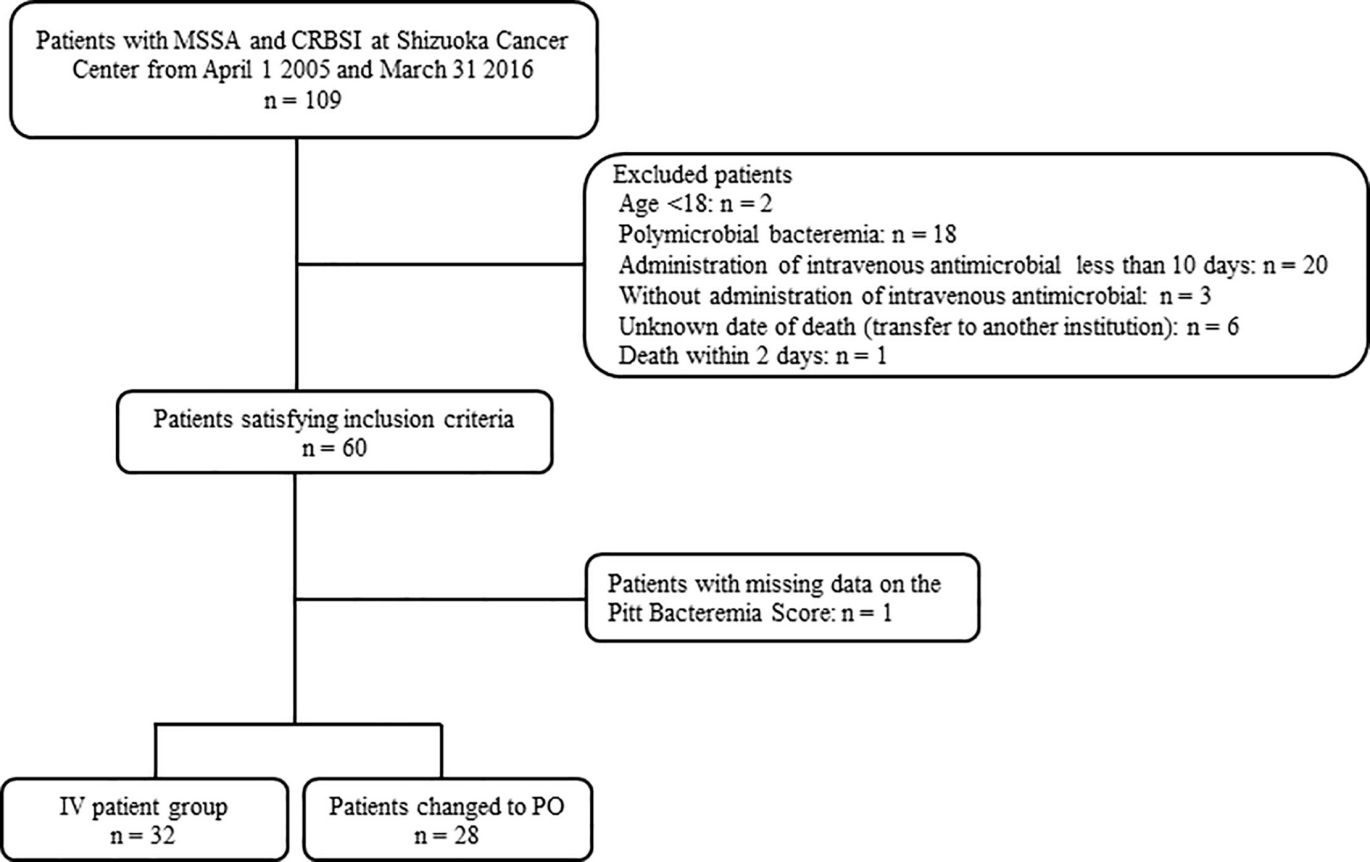
Flow diagram of patients included in the study. Abbreviation: MSSA, methicillin-sensitive *Staphylococcus aureus*; CRBSI, catheter-related bloodstream infection; IV, intravenous; PO, oral.

A total of 109 patients were screened for inclusion; of these, 49 were excluded (Fig 1) [one patient with polymicrobial bacteremia also being less than 18 years of age]. One case had missing data for the Pitt Bacteremia Score. Our study included a final total of 60 patients (IV group, n = 32 [53.3%]; IV + PO group, n = 28 [46.7%]). Table 1 shows the characteristics of patients in each group. The median (IQR) age of the patients was 60 (range, 50–71.0) years. The distribution of place of acquisition was significantly different between the two groups: while hospital onset was higher in the IV group, community-acquired and health care-associated were higher in the IV + PO group (p = 0.039). Significant differences occurred in the rate of thromboembolism (IV group, n = 0 [0.0%]; IV + PO group, n = 6 [21.4%]; p = 0.008), ID consultation (IV group, n = 21 [65.6%]; IV + PO group, n = 26 [92.9%]; p = 0.013), received echocardiogram (IV group, n = 14 [43.8%]; IV + PO group, n = 24 [85.7%]; p = 0.001), and intravenous vancomycin (IV group, n = 23 [71.9%]; IV + PO group, n = 27 [96.4%]; p = 0.014). Furthermore, the median durations of therapy (IV group, 17 days [13–31 days]; IV + PO group, 33 days [26–52 days]; p<0.001) were significantly different between the two groups. Recurrence within 30 days of treatment did not show any statistical differences between the two groups (IV group, n = 1 [3.13%]; IV + PO group, n = 0; p>0.999). Both groups had no infection-related death.

**Table 1:** Comparison of demographic and clinical characteristics between IV and IV + PO groups.

		All (n = 60)	IV group (n = 32)	IV+PO group (n = 28)	P-value	
**Age (median, IQR)**		60.0(50.0, 71.0)	62.5(58.0, 71.0)	57.5(44.3, 70.0)	0.060	a
**Male gender**		35(58.33%)	20(62.50%)	15(53.57%)	0.601	b
**Place of acquisition**					0.039	b
	**Community-acquired**	1(1.67%)	0(0.00%)	1(3.57%)		
	**Hospital onset**	39(65.00%)	25(78.13%)	14(50.00%)		
	**Healthcare-associated**	20(33.33%)	7(21.88%)	13(46.43%)		
**Hematologic malignancy**		4(6.67%)	2(6.25%)	2(7.14%)	>0.999	b
**Solid tumor**		56(93.33%)	30(93.75%)	26(92.86%)	>0.999	b
**Types of neoplasm**					0.543	b
	**Lip, oral cavity, and pharynx**	7(11.67%)	6(18.75%)	1(3.57%)		
	**Digestive organs**	30(50.00%)	16(50.00%)	14(50.00%)		
	**Respiratory and intrathoracic organs**	5(8.33%)	3(9.38%)	2(7.14%)		
	**Bone and articular cartilage**	2(3.33%)	0(0.00%)	2(7.14%)		
	**Mesothelial and soft tissue**	3(5.00%)	1(3.13%)	2(7.14%)		
	**Female genital organs**	2(3.33%)	1(3.13%)	1(3.57%)		
	**Urinary tract**	3(5.00%)	2(6.25%)	1(3.57%)		
	**Ill-defined, other secondary, and unspecified sites**	4(6.67%)	2(6.25%)	2(7.14%)		
	**Others**	4(6.67%)	1(3.13%)	3(10.71%)		
**Organ transplantation**		0(0.00%)	0(0.00%)	0(0.00%)		
**Immunosuppressant**		0(0.00%)	0(0.00%)	0(0.00%)		
**Glucocorticoid therapy**		7(11.67%)	2(6.25%)	5(17.86%)	0.235	b
**Total parenteral nutrition**		27(45.00%)	16(50.00%)	11(39.29%)	0.446	b
**Antineoplastic chemotherapy**		45(75.00%)	24(75.00%)	21(75.00%)	>0.999	b
**Diabetes mellitus**		14(23.33%)	8(25.00%)	6(21.43%)	0.770	b
**Chronic kidney disease**		3(5.00%)	3(9.38%)	0(0.00%)	0.241	b
**Neutropenia**		1(1.85%)	1(3.33%)	0(0.00%)	>0.999	b
**Hemodialysis**		2(3.33%)	2(6.25%)	0(0.00%)	0.494	b
**Valve disease**		4(6.67%)	3(9.38%)	1(3.57%)	0.616	b
**Type of CVCs**					0.064	b
	**Short-term central venous catheter**	23(38.33%)	16(50.00%)	7(25.00%)		
	**PICC**	1(1.67%)	0(0.00%)	1(3.57%)		
	**Implanted port**	36(60.00%)	16(50.00%)	20(71.43%)		
**Presence of a non-removable foreign body**					0.402	b
	**IV filter**	1(1.67%)	0(0.00%)	1(3.57%)		
	**None**	56(93.33%)	31(96.88%)	25(89.29%)		
	**Others**	3(5.00%)	1(3.13%)	2(7.14%)		
**Pitt Bacteremia Score**					0.841	a
**0**		31(52.54%)	16(51.61%)	15(53.57%)		
**1**		19(32.20%)	10(32.26%)	9(32.14%)		
**2**		1(1.69%)	1(3.23%)	0(0.00%))		
**3**		2(3.39%)	1(3.23%)	1(3.57%)		
**4**		4(6.78%)	1(3.23%)	3(10.71%)		
**8**		2(3.39%)	2(6.45%)	0(0.00%)		
**Secondary foci**						
	**Postoperative wound**	1(1.67%)	1(3.13%)	0(0.00%)	>0.999	b
	**Pneumonia**	1(1.67%)	0(0.00%)	1(3.57%)	0.467	b
	**Skin soft tissue**	1(1.67%)	1(3.13%)	0(0.00%)	>0.999	b
	**Endocarditis**	1(1.67%)	1(3.13%)	0(0.00%)	>0.999	b
	**Thromboembolism**	6(10.00%)	0(0.00%)	6(21.43%)	0.008	b
	**Liver abscess**	1(1.67%)	0(0.00%)	1(3.57%)	0.467	b
**Persistent bacteremia**		6(20.00%)	1(7.69%)	5(29.41%)	0.196	b
**Removed CVCs**		55(91.67%)	27(84.38%)	28(100.00%)	0.055	b
**Drainage except for removing CVCs**		5(8.33%)	2(6.25%)	3(10.71%)	0.657	b
**CVCs reinsertion during antibiotic treatment**		24(40.00%)	15(46.88%)	9(32.14%)	0.297	b
**Complication after treatment**						
	**Thromboembolism**	5(8.33%)	1(3.13%)	4(14.29%)	0.175	b
	**Osteomyelitis**	1(1.67%)	0(0.00%)	1(3.57%)	0.467	b
	**Skin soft tissue**	1(1.67%)	0(0.00%)	1(3.57%)	0.467	b
	**None**	55(91.67%)	31(96.88%)	24(85.71%)	0.175	b
**ID consultation**		47(78.33%)	21(65.63%)	26(92.86%)	0.013	b
**Received echocardiogram**		38(63.33%)	14(43.75%)	24(85.71%)	0.001	b
**IV antibiotics**						
	**CEZ**	56(93.33%)	29(90.63%)	27(96.43%)	0.616	b
	**CMZ**	3(5.00%)	2(6.25%)	1(3.57%)	>0.999	b
	**CTRX**	4(6.67%)	3(9.38%)	1(3.57%)	0.616	b
	**CFPM**	24(40.00%)	9(28.13%)	15(53.57%)	0.065	b
	**SBT/CPZ**	5(8.33%)	4(12.50%)	1(3.57%)	0.359	b
	**SBT/ABPC**	6(10.00%)	3(9.38%)	3(10.71%)	>0.999	b
	**TAZ/PIPC**	12(20.00%)	5(15.63%)	7(25.00%)	0.520	b
	**IPM/CS**	3(5.00%)	1(3.13%)	2(7.14%)	0.594	b
	**MEPM**	3(5.00%)	2(6.25%)	1(3.57%)	>0.999	b
	**VCM**	50(83.33%)	23(71.88%)	27(96.43%)	0.014	b
	**CLDM**	6(10.00%)	2(6.25%)	4(14.29%)	0.404	b
	**others**	2(3.34%)	2(6.25%)	0(0.00%)		
**PO antibiotics**						
	**CEX**	19(31.67%)		19(67.86%)		
	**CVA/AMPC**	4(6.67%)		4(14.29%)		
	**LVFX**	4(6.67%)		4(14.29%)		
	**ST**	2(3.33%)		2(7.14%)		
	**CLDM**	6(10.00%)		6(21.43%)		
	**others**	2(3.33%)		2(7.14%)		
**Duration of antibiotics days (median, range)**		26.0(15,0, 34.0)	16.5(13.0,30.5)	32.5(26.0,52.3)	<0.001	b
**Duration of IV antibiotics (median, range)**		17.0(13.0,24.75)	16.5(13.0,30.5)	17.5(13.0,24.0)	0.935	b
**Recurrence**		1(1.67%)	1(3.13%)	0(0.00%)	>0.999	b
**Infection-related death**		0(0.00%)	0(0.00%)	0(0.00%)		
**Death within 90 days**		20(33.33%)	17(53.13%)	3(10.71%)	0.001	b
**Death within 30 days**		7(11.67%)	7(21.88%)	0(0.00%)	0.012	b

data: n, %; median [interquartile range (IQR): 25%, 75%].

a, Mann-Whitney U test

b, Fisher's Exact Test.

Abbreviations; IV, intravenous; PO, oral; IQR, interquartile range; CVCs, central venous catheters; PICC, peripherally inserted central catheter; ID, infectious disease; CEZ, Cefazolin; CMZ, Cefmetazole; IPM/CS, Imipenem/Cilastatin; MEPM, Meropenem; VCM, vancomycin; CLDM, Clindamycin; CEX, Cefalexin; CVA/AMPC, Amoxicillin/Clavulanate; LVFX, Levofloxacin; ST, Sulfamethoxazole/Trimethoprim.

The 90-day mortality in the IV and IV + PO groups were 53.1% (17/32) and 10.7% (3/28), respectively (p = 0.001). The 30-day mortality in the IV and IV + PO groups were 21.9% (7/32) and 0% (0/28), respectively (p = 0.012). The propensity score was estimated using multivariable logistic regression analysis with the following variables: age, gender, place of acquisition, Pitt Bacteremia Score, glucocorticoid therapy, antineoplastic chemotherapy, diabetes mellitus, hematologic malignancy, solid tumor, types of neoplasm, chronic kidney disease, hemodialysis, valve disease, presence of non-removable foreign body, ID consultation, and duration of therapy of more than 1 month. Compared to the IV group, the odds ratios of 90-day mortality in the IV + PO group was 0.106 (95% CI: 0.027–0.423; p = 0.001) (Table 2). In the propensity score-adjusted multivariate logistic regression model, compared to the IV group, the odds ratios of 90-day mortality in the IV + PO group was 0.377 (95% CI: 0.037–3.884; p = 0.413).

**Table 2:** Univariate and propensity score-adjusted analysis of risk factors associated with mortality within 90 days.

	Alive (n = 40)	Death (n = 20)	Unadjusted OR (95% CI)	P-value	PS-adjusted OR (95% CI)	P-value
**IV group**	15(37.50%)	17(85.00%)	ref.			
**IV + PO group**	25(62.50%)	3(15.00%)	0.106(0.027–0.423)	**0.001**	0.377(0.037–3.884)	0.413

OR: odds ratio, 95% CI: 95% confidence interval.

Abbreviations: PS, Propensity score; IV, intravenous; ref, reference; PO, oral.

## Discussion

This study compared the 90-day mortality of IV therapy with IV + PO therapy in cancer patients with CRBSI due to MSSA, and provides the first known evidence of the efficacy of oral switch therapy in cancer patients with CRBSI due to MSSA. The findings of the present study suggest that oral switch therapy might be a reasonable option for this patient population, in whom prolonged intravenous therapy can often be difficult to implement. In the propensity score-adjusted multivariate logistic regression model using variables likely to influence the outcomes, no significant association was found with mortality.

The univariate logistic regression model showed that mortality in patients who switched to oral therapy (IV + PO group) was significantly lower than that in the IV treatment group. The switch might have confounded our findings since current guidelines do not recommend oral switch therapy [3,4]. We considered that the presence of confounding factors might have influenced mortality in our study, and thus performed multivariate analysis; however, the adjusted mortality was not significantly different between groups. Although many experts have considered that IV treatment is necessary for this patient population, our findings showed that switching to oral therapy was not associated with poorer outcomes in cancer patients with CRBSI due to MSSA. Therefore, the results of the present study are quite novel.

Previous studies have shown that adherence to evidence-based quality-of-care indicators (QCIs), as well as ID consultation, which is associated with adherence to QCIs in the management of SAB, could result in reduced mortality [2,14,15]. The main QCIs consist of the following five aspects: (1) follow-up blood cultures; (2) early source control when applicable; (3) echocardiography; (4) the early use of appropriate antibiotics, and (5) the appropriate duration of therapy [15]. In our study, there was a low adherence rate to follow-up blood cultures, echocardiography, and removal of CVCs in the IV group compared to the proposed QCIs. However, some reports do not recommend routine echocardiography [16–18], and performing routine echocardiography for SAB is controversial. Only 84% of patients in the IV group had the CVCs removed, which was primarily due to consideration of their cancer prognosis.

However, ID consultation, which adheres to QCIs, has also been shown to be associated with reduced mortality [2]. In this study, it is not known why oral switch therapy was adopted in certain patients, and this may have led to an unknown bias. However, it is possible that oral switch therapy could be an option for patients with CRBSI due to MSSA if they adhere to QCIs and ID consultation.

Pharmacist-facilitated antimicrobial stewardship initiatives can aid in switching from IV to PO [19]. While IV medications may be more bioavailable and have greater effects, some oral drugs produce serum levels comparable to the parenteral form [5]. In our study, cefalexin was the most common oral antibiotic option. The antimicrobial activity of cephalexin has been shown to be active against common Gram-positive organisms, including MSSA [20]. Furthermore, orally-administered cephalexin has been shown to be almost completely absorbed from the gastrointestinal tract [20].

The mortality in our study was significantly higher than that in previous studies, particularly in patients on IV treatment [2]. This finding might have resulted from the fact that all participants in the present study were cancer patients. In some studies, cancer is reported to be a predictor of mortality in *S*. *aureus* bacteremia [21–23]. Although the design of our study did not provide an opportunity to determine the weight of this association, we did evaluate variables related to neoplastic disease that could explain this outcome.

The recurrence of MSSA infections complicated by bacteremia was similar between the two treatment groups in our study. Our findings were similar to those of a meta-analysis that included 11 studies, which showed that the relapse rate after conventional 4- to 6-week antibiotic regimens for catheter-related *S*. *aureus* bacteremia is expected to be less than 1% [1].

The present study has several limitations. First, a relatively small number of patients were enrolled in the study. Second, because the study used data from retrospective medical records review, the patients were not treated according to defined protocol. Thus, type of antibiotics, dose, and duration of treatment differed in our participants. In addition, echocardiography and follow-up blood culture were not performed in all patients; therefore, the true incidence of complications and recurrences may have been underestimated. Additionally, there were some missing data in our study; however, rigorous and consistent data collection and verification were ensured by four ID doctors. Likewise, both groups had no infection-related death in our study. The reason for this finding is unclear, and we considered this as a limitation of retrospective data collection. Therefore, we evaluated 30-day mortality in addition to 90-day mortality.

Third, data were collected at a single institution treating cancer patients with CRBSI, and the predominant places of acquisition of infection were hospital onset and health care-associated infections; hence, our results may not be generalizable. Our results should therefore be validated in other cohorts, especially in non-cancer patients. Finally, there was a potential for confounding by indication. The majority of patients, at the time of admission to the hospital for severe infections, often tend to opt for IV medications and continue to receive them until discharge. Patients receiving IV antibiotics might differ from patients receiving oral antibiotics after IV treatment in ways that could have been associated with mortality. Furthermore, cancer staging and performance status, which might have influenced mortality, were not obtained in our study. However, patient characteristics were not significantly different between groups. We attempted to limit the potential for unadjusted confounding by including both measures of illness severity based on vital signs at baseline (i.e., Pitt Bacteremia Score) and comorbidities.

In conclusion, our study suggests that CRBSI due to MSSA in cancer patients can be treated by switching to oral therapy, rather than relying on conventionally recommended IV therapy. Future prospective randomized trials are needed to better clarify our findings and confirm whether switching to oral therapy for CRBSI due to MSSA is indicated in cancer patients.

## Supporting information

S1 File Raw data of our patients. (XLSX)
